# The mediating role of scientifical-medical satisfaction between COVID-19 conspiracy beliefs and vaccine confidence: a two-waves structural equation model

**DOI:** 10.1007/s10865-022-00322-5

**Published:** 2022-06-22

**Authors:** Giuseppe Mignemi, Anna Panzeri, Umberto Granziol, Giovanni Bruno, Marco Bertamini, Giulio Vidotto, Andrea Spoto

**Affiliations:** Department of General Psychology, University of Padua, via Venezia 8, 35131 Padova, Italy

**Keywords:** COVID-19, Longitudinal structural equation models, Vaccine confidence, Conspiracy beliefs

## Abstract

Vaccine confidence has emerged as one of the most relevant psychological factors implied in the worldwide affecting the fight against COVID-19—as well as public trust in doctors, medicine, and science. Indeed, the vaccine confidence is crucial to maximize the trust in vaccines and their use for prevention, with several implications for public health. This study aimed to analyse the relationships among between vaccine confidence, conspiracy beliefs about COVID-19, and satisfaction with science and medicine in handling the COVID-19 pandemic. A longitudinal observational survey was administered to a convenience sample (n = 544; mean age 52.76 y.o., SD = 15.11; females 46.69%) from the Italian general population. A two-waves mediation model—a structural equation model technique—was used. The survey was part of a larger international project (https://osf.io/qy65b/). The model highlighted that the conspiracy beliefs about COVID-19 had a negative effect on the satisfaction with medicine and science (β = − 0.13, se = 0.03, *p* < .001). The latter, in turn, had a positive effect on vaccine confidence (β = 0.10, se = .05, *p* < .001). Interestingly, the effect of conspiracy beliefs on vaccine confidence was completely mediated by the scientifical-medical satisfaction (β = − 0.02, se = 0.01, *p* < .05). These results highlight how the scientifical-medical satisfaction can fully mediate the relationship between conspiracy beliefs about COVID-19 and vaccine confidence. These findings about vaccine hesitancy and confidence and disclose have implications for psychological and social interventions that could promote vaccine confidence by targeting the satisfaction with science and medicine.

## Introduction

Almost two years after its first appearance, the COVID-19 pandemic continues to affect the health of millions of individuals, leading in many cases to death or to long-term issues (Li et al., 2021). Importantly, COVID-19 also represents a challenge to psychological health (WHO, 2021), with many studies describing the negative consequences in terms of fear, anxiety, and depression among healthcare workers (Benfante et al., 2020; Panzeri, Bertamini, et al., 2021; Panzeri, Rossi Ferrario, et al., 2021), the general (Epifanio et al., 2021; Parola et al., 2020) and clinical population (Panzeri & Rossi Ferrario, 2020; Rossi et al., 2021; Rossi Ferrario et al., 2021). Ongoing medical research is trying to contain its spread but the long-term consequences are still largely unknown. To date, vaccines are the most effective containment measure. They create antibodies that contrast the virus once in contact with the human organism (Li et al., 2021). However, consulting a physician is recommended to evaluate the pros and cons of vaccination for everyone’s clinical condition to balance the individual risks and benefits. A minority of people cannot be vaccinated for medical reasons (e.g., pre-existing conditions such as allergies, diseases, etc.). These people need to be protected by encouraging vaccinations among those who are suitable.

Number of research studies proved the efficacy of vaccines in preventing and reducing the COVID-19 manifestations, thus allowing: (1) absence of symptoms, (2) reduced stress on the organism, (3) reduce risk of contagion, (4) reduced burden on the health institutes and facilities, (5) protection of fragile individuals (e.g., elderlies, immunosuppressed, with comorbidities), (6) and saving more lives (ISS, 2021). Thus, it is important that the vaccine adoption is wide, as the WHO, the ISS, and other international organizations have claimed.

Despite the several advantages of vaccines, both for self and society, some people are reluctant to get vaccinated, with a negative impact on the individual and public health, as well as for society and the economy (Romer & Jamieson, 2020; Sallam, 2021). So, why do some people refuse to receive the vaccine? Why do some think that it is better not to get vaccinated?

The choice not to get vaccinated is also referred to as vaccine hesitancy, or *low vaccine confidence* (VC). It is a complex issue with several motivations behind (Murphy et al., 2021; Pivetti et al., 2021). Indeed, *vaccine confidence* may be conceived as the outcome of a number of variables influencing it. Among the most important factors influencing vaccine confidence, there are conspiracy beliefs related to the COVID-19 pandemic that are akin to the analogous conspiracy theories (Douglas, 2021; Sturgis et al., 2021). Some of them are expressed with these words: *“Vaccines are useful only to enrich the big-pharma institutes and the physicians”, “Vaccines are no more effective than other alternative methods”, “The vaccine inoculates a microchip to control us…”, “They want to control us, to tell us what to do”*.

*Conspiracy beliefs* rely on a common basis that is a conspiracy view of the world—a mechanism to justify a system (Jost et al., 2004). Conspiracy theories are powerful because they can justify and explain complex phenomena in a simple way. In other words, conspiracy beliefs can provide a simple explanation and are boosted by the need to have simple certainties. As an example, in front of a threatening, complex and global event—as the COVID-19 pandemic—people try to provide various explanations. In particular, people with a sense of lack of control in their lives, do not feel responsible and are more prone to think that the responsibility of a negative event relies on the social classes that they think have the control (Grzesiak-Feldman, 2013). Interestingly, conspiracy theories are often sustained by groups not feeling part of a community and feeling excluded by the society or outside it. Such external groups may be both of higher and lower social status, indeed, people with higher social status may fear that the present social order could change.

The distrust toward others underlying the conspiracy theories is learned through life experiences for long years (e.g., family, society, culture), thus are rigid, well-built, and very difficult to modify. Despite mistrust may have been useful in certain contexts, in other circumstances may be a disadvantage (Romer & Jamieson, 2020)—such as when deciding to get vaccinated or not.

In addition to the conspiracy beliefs that prevent vaccination, there is another important factor to consider, the low *Scientifical-Medical Satisfaction* (SMS)—including the national health system, doctors, nurses, scientists, and researchers. Scientists and researchers are viewed as distant, and they speak a difficult language. They are also held responsible for not having done more to prevent the spread of COVID-19. Conspiracy beliefs may have a role in lowering SMS. In this regard, some people think that scientists only obey the ruling classes (or to the so-called “big powers”) but do not really care for people’s health (Pivetti et al., 2021; Sturgis et al., 2021). Also, the public health system is perceived as very close to and highly influenced by the political forces (also perceived as negative), thus science and medicine are once again perceived as more distant, unknown, and not controllable.

However, to date, evidence about the low vaccine confidence in Italy is scarce and there are few longitudinal studies on this topic (Fridman et al., 2021; Lee & Sibley, 2020). Thus, it is not clear how these complex factors may interact with each other in leading to a lower or higher vaccine confidence.

In view of this background, we expect to observe a pattern. On one hand, for some people with *conspiracy beliefs* and low levels of SMS, the credibility of vaccines and their producers is questionable, thus leading to low *vaccine confidence*. On the other hand, people with lower conspiracy beliefs and higher SMS, are expected to have higher vaccine confidence.

**Fig. 1:**
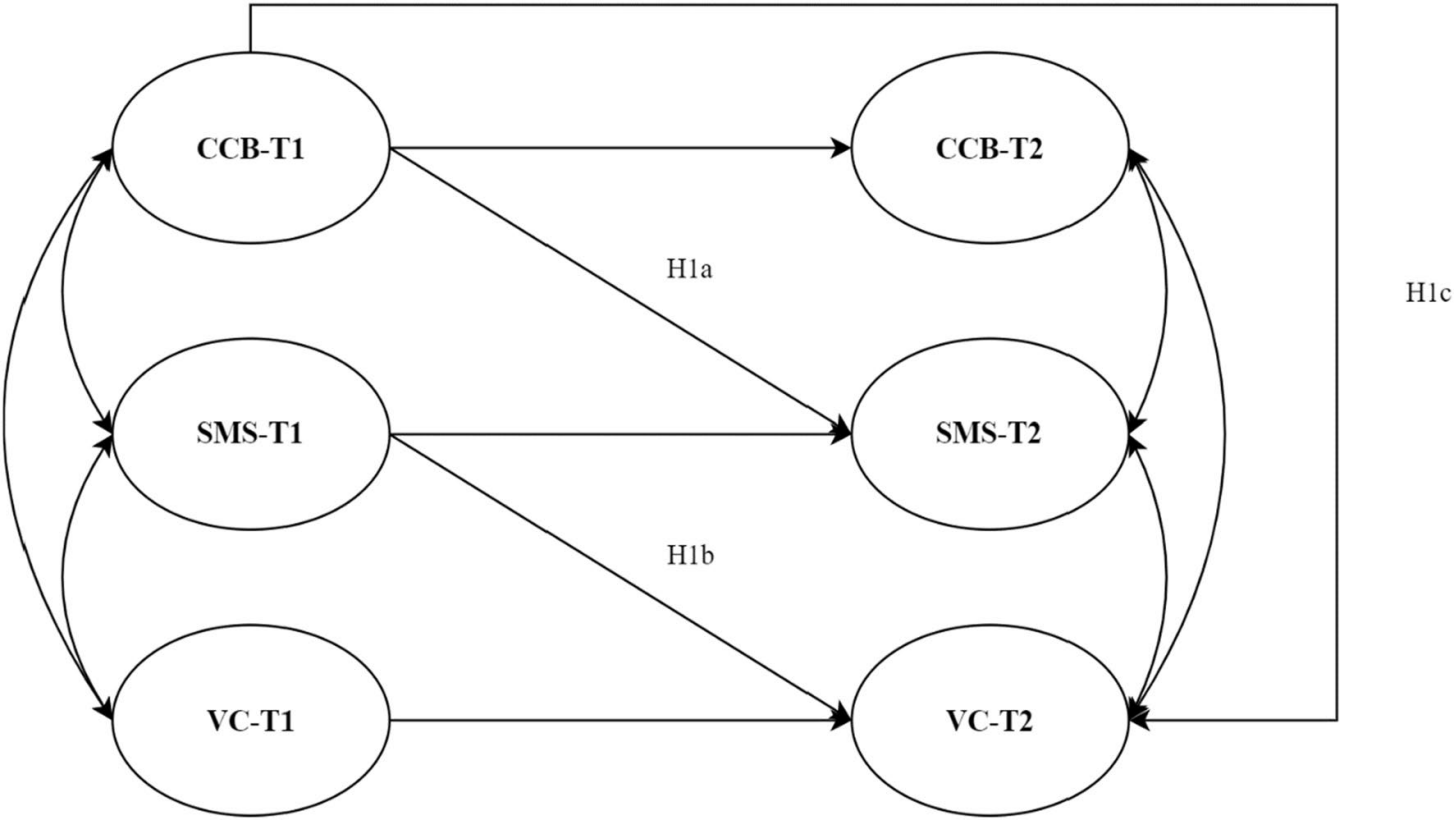
Model Hypotheses. H2 was removed from the diagram for sake of simplicity: it is the indirect effect of CCB-T1 on VC-T2 estimated as product of the path regression coefficients H1a and H1b. Curved double arrow stands for correlation between two different variables; non-curved arrow stands for regression coefficient. Straight arrow that goes from a variable at T1 to the same variable at T2 stands for autoregressive coefficient (the change of the variable from T1 to T2)

Thus, this longitudinal study aimed to understand the relationship between vaccine confidence, COVID-19 conspiracy beliefs (CCB) and SMS, by provide a possible model to explain vaccine confidence (Fig. 1). More precisely, it was hypothesized that high levels of CCB at T1 are predictive of low levels of SMS at T2 (H1a), high levels of SMS at T1 are predictive of high levels of VC at T2 (H1b), CCB has a direct (H1c) and mediated effect on VC (H2). Thus, it was a two-waves mediation model (Cole & Maxwell, 2003; Little, 2013). Therefore, stationarity (i.e., “the degree to which one set of variables produces change in another set remains the same over time”, Cole & Maxwell, 2003, p. 560) and the temporal precedence of H1a path with respect to H1b path were assumed (Little, 2013).

## Methods and materials

### Procedure

We conducted a longitudinal study with two waves. The first data collection took place after the first contagion peak (end of March 2020) and the end of the first strict national lockdown (May 2020). The second data collection took place nine months later, after the second easing of restrictive measures (e.g., curfew, red zone). The first wave data collection was from July 13th to July 28th, 2020, the second one was from April 30th to May 26th, 2021.

An online survey was developed and administered using the Qualtrics platform for both waves. The procedures and the survey were identical in the two measurement occasions. The median time of completion of the survey was roughly 42 min for the first wave and 45 min for the second wave. After that each participant was reimbursed by Qualtrics™.

The survey was administered in four of the most populous Italian regions—Campania, Lazio, Lombardia and Veneto. The inclusion criteria were: (a) living in one of the four regions, (b) to be at least 18 years old. All participants provided informed consent before completing the survey of the study, conducted according to Ethical Principles and Code of Conduct of the Italian Association of Psychology. Ethical approval was provided by the Ethical Committee for Psychological Research of the University of Padua (protocol number: 3818).

### Participants

The inclusion criteria were: (a) living in one of the previous regions, (b) to be at least 18 years old. All participants provided informed consent before completing the survey of the study, conducted according to Ethical Principles and Code of Conduct of the Italian Association of Psychology. Ethical approval was provided by the Ethical Committee for Psychological Research of the University of Padua.

544 adult participants were recruited for this study by Qualtrics from an online research panel. A stratified quota sampling was used to guarantee the right match between the Italian population and the sample characteristics (gender, age, household income, and region).

The mean age of the sample was 52.77 years (median = 55, SD = 15.11, range = 18–87), and 46.69% were female (n = 254). Participants were enrolled from the four selected regions based on their population size: Campania (20.95%, n = 121), Lazio (25.55%, n = 131), Lombardia (37.68%, n = 207), Veneto (15.80%, n = 85). Most of them were Italian (96.69%, n = 526) and with Caucasian ethnicity (77.02%, n = 419). Nearly half of the sample had completed high school (57.53%, n = 313) and a further 42.47% having attained a higher level of education. Less than half were in full employment (43.75%, n = 238), with 24.18% retired (26.65%, n = 145).

All participants completed the survey at the first and at the second time data collection.

### Measures

As part of an international consortium (McBride et al., 2021), the measures of the present study were drawn from the Italian C19PRC study (Bruno et al., 2020). In particular, sociodemographic variables (i.e., gender, age, household income, and residence region) and the following measures were collected:

*COVID-19 related Conspiracy Beliefs* (CCB): in line with other studies carried out in the international consortium (e.g., Gibson-Miller et al., 2020; McBride et al., 2021), 3 visual analogue scales (VAS) from 0 (= ‘I do not believe it at all’) to 100 (= ‘I believe it totally’) were used to assess the individual adherence to COVID-19 related conspiracy beliefs (e.g., “The 5G networks are the real responsible of the current pandemic”). It showed an acceptable internal consistency (T1: McDonald’s ω = 0.61, T2: McDonald’s ω = 0.62).

*Scientifical-Medical Satisfaction* (SMS): 3 visual analogue scales (VAS) from 0 (= ‘Not satisfied at all’) to 100 (= Completely satisfied) were used to assess the individual satisfaction with the work done by the national health system, doctors, and scientist. An example of the item was “Overall, how satisfied is the work of scientists in responding to the pandemic by COVID-19”. It showed a good internal consistency in both measurement occasions (T1: McDonald’s ω = 0.87; T2: McDonald’s ω = 0.92).

*Vaccines Confidence* (VC): the individual general vaccine confidence was assessed by 10 items adapted from McBride et al. (2020) on a 5-point Likert scale from 1 (= “Strongly disagree”) to 5 (= “Strongly agree”). Examples of items were “I am generally in favour of vaccination” and “Vaccination of healthy people helps to protect others, stopping the contagion of the disease”. It showed a good internal consistency in both measurement occasions (T1: McDonald’s ω = 0.89; T2: McDonald’s ω = 0.89).

Psychometric properties and summary statistics of the scales are reported in Table 1.

**Table 1:** Psychometric Properties of the scales

Latent variable and reflective indicators	*m* (*SD*)	Sk	K	*λ*			*ω*	AVE
				Mod. C	Mod. W	Mod. S		
*CCB T1*							0.61	0.35
CCB1	43.1 (34.9)	0.14	− 1.37	0.99***	0.99***	0.99***		
CCB2	13.4 (22.8)	1.95	2.97	1.12***	0.99***	0.99***		
CCB3	24.1 (29.3)	1.08	− 0.04	0.88***	1.01***	1.01***		
*CCB T2*							0.62	0.36
CCB1	44.6 (33.8)	0.10	− 1.27	0.93***	0.99***	0.99***		
CCB2	12.7 (21.4)	1.88	2.72	0.91***	0.99***	0.99***		
CCB3	23.4 (29.3)	1.13	0.07	1.14***	1.01***	1.01***		
*SMS T1*							0.87	0.71
SMS1-T1	77.7 (23.2)	− 1.42	1.68	0.99***	0.99***	0.99***		
SMS2-T1	73.6 (23.9)	1.14	0.90	1.11***	1.09***	1.09***		
SMS3-T1	73.5 (23.6)	− 0.98	0.48	0.89***	0.91***	0.91***		
*SMS T2*							0.92	0.80
SMS1-T2	73.5 (24.5)	− 1.22	0.92	0.99***	0.99***	0.99***		
SMS2-T2	69.3 (25.4)	− 1.00	0.27	1.08***	1.09***	1.09***		
SMS3-T2	71.2 (25.4)	− 1.08	0.47	0.93***	0.91***	0.91***		
*VC T1*							0.89	0.62
pVC1-T1	3.89 (0.83)	− 0.80	− 0.01	0.91***	0.90***	0.90***		
pVC2-T1	3.59 (0.89)	− 0.57	− 0.26	1.06***	1.09***	1.09***		
pVC3-T1	3.51 (0.83)	− 0.64	0.43	0.97***	0.99***	0.99***		
pVC4-T1	3.56 (0.99)	0.14	− 0.79	1.11***	1.08***	1.08***		
pVC5-T1	3.50 (0.91)	− 0.03	− 0.65	0.94***	0.95***	0.94***		
*VC T2*							0.89	0.62
pVC1-T2	4.08 (0.86)	− 0.58	− 0.70	0.88***	0.90***	0.90***		
pVC2-T2	3.67 (0.97)	− 0.39	− 0.42	1.11***	1.09***	1.09***		
pVC3-T2	3.63 (0.90)	− 0.26	− 0.41	1.01***	0.99***	0.99***		
pVC4-T2	3.46 (1.08)	− 0.06	− 0.95	1.05***	1.08***	1.08***		
pVC5-T2	3.48 (1.01)	− 0.21	− 0.56	0.94***	0.95***	0.94***		

*Note: CCB* COVID-19 related conspiracy beliefs, *SMS* Scientifical-medical satisfaction, *VC* Vaccine confidence, *T1* Time 1, *T2* Time 2, *Model T1* Model for the single measurement construct, *Model-C* Model in which configural invariance was checked, *Model-W* Model in which weak invariance was checked, *Model-S* Model in which strong invariance was checked, *p(…)* Item parcel, *m* Mean, *SD* Standard deviation, *Sk* Skewness, *K* Kurtosis, *ω*, McDonald’s Omega, *AVE* Average variance extracted, *C19* COVID-19. *p* < .05; ** *p* < .01; *** *p* < .001

### Data analysis

All analyses were performed with the *R* statistical software system: R-core project (R Core Team, 2021). The following packages were used: *lavaan* (v.0.6-6; Rosseel, 2012) MVT (v.5.8; Maintainer & Osorio, 2015; Korkmaz et al., 2014), *semTools* (v.0.5-4; Jorgensen, 2018), and *influence.SEM* (Pastore & Altoè, 2018).

### Preliminary analysis

In a preliminary step, the presence of missing data (i.e., nonresponse, attritions) was assessed (Little, 2013). A descriptive analysis was conducted to verify the empirical distribution of the data within and between the two data collection waves. A correlation matrix was computed to verify the association among the items. Variance Inflation Factor (VIF) was computed for each predictor variables using raw score in order to verify potential multicollinearity among variables (Consoli et al., 2020; Tabachnick and Fidell, 2007).

### Reliability, common method bias and parceling

The reliability of the single scales was separately assessed for each measurement occasion. For each scale McDonald’s ω and Average Variance Extracted (AVE) index were computed.

The possible presence of common method bias (Bagozzi and Yi, 1991) was checked with the Harman Single Factor test (Harman, 1976; Podsakoff et al., 2003). A model in which all the items were loaded onto a first-order single latent factor was specified. An alternative model with 6 first-order correlated latent factors (3 *per* measurement occasion: CCB-T1, SMS-T1, VC-T1, CCB-T2, SMS-T2, VC-T2) and 3 s-order factors was specified (the first-order factors of T1 and T2 measures of each construct were specified to load onto a second-order factor to account for the shared variance due to same measured construct: CCB-T1 and CCB-T2 onto the same second-order factor, VC-T1 and VC-T2 onto the same second-order factor, and SMS-T1 and SMS-T2 both onto another second-order factor as well). Based on the empirical distribution, the Mardia test (skewness: γ^1^, *p* = 47.71, χ^2^_(1330)_ = 2392, *p* < .001; kurtosis γ^2^, *p* = 506.11, z = 44.98, *p* < .001), and the nature of the response scales a diagonally weighted least square (DWLS) estimator was used for each model of this study.

The models were then compared using the test differences in goodness-of-fit indices (Δχ^2^: *p* < *.050*; comparative fit index difference ΔCFI: > 0.010; root mean square error of approximation (RMSEA) difference ΔRMSEA: > 0.015). A statistically significant chi-square difference (Δχ^2^; *p* < .05) and a CFI difference greater than 0.01 provide evidence of the absence of common method bias (Brown, 2015; W. G. Cheung & Rensvold, 2002; A. Rossi et al., 2020). Due to violation of multivariate-normality of the data a Satorra-Bentler Scaled Chi-Squared Difference Test was used for all the model comparisons of the study (Satorra and Bentler, 2001).

For each model tested a ratio *N*:*q* of 7 participants per free parameter was ensured (Bentler & Chou, 1987).

A balancing parceling approach (Little, 2013) was used for the 10 items of the vaccine confidence scale (the item with the highest factor loading is paired with the item that has the lowest one, the second with the second-last and so on).

### Measurement model and invariance analysis

Before testing the hypothesized structural model, a Confirmatory Factor Analysis (CFA) was performed for each scale to assess the structural validity of the measurement model. Every scale was separately tested in a single model and compared with alternative models in which different numbers of latent factors were specified. In this step (before the invariance analysis) all the three scales administered in two measurement occasions were tested separately.

To evaluate the goodness of fit of the model, the following fit indices were used: χ^2^, RMSEA, CFI, Standardized Root Mean Square Residual (SRMR). Acceptable model fit was defined based on the recommended cut-off values: χ^2^ non-statistically significant; χ^2^/*df* < 3; RMSEA less than 0.06, 0.06 to 0.08 for a reasonable fit; CFI greater than 0.95, 0.90 to 0.95, for a reasonable fit; SRMR less than 0.08 (Iacobucci, 2010; Kline, 2015; Marsh et al., 2004).

To perform a two-waves mediation model with a Structural Equation Modelling (SEM) approach with latent variables, an invariance analysis was performed for all the scales. The configural (i.e., pattern invariance; no constraints were imposed to any of the parameters of the model), the weak (i.e., metric invariance; each corresponding loading was constrained to be equal across measurement occasions) and the strong (i.e., scalar invariance; each corresponding intercept was imposed to be equal across measurement occasions) invariances were assessed for these scales. For each model in which repeated assessment of the same indicators are present (i.e., the same scale at two different measurement occasions) a longitudinal null model was specified. In the latter all the covariances among the observed variables were fixed to 0, and the respective variances and means of the indicators were constrained to be equal across different measurement occasions (Little, 2013; Widaman and Thompson, 2003). All the fit indices were computed having as null model the respective longitudinal null model. A non-statistically significant chi-square difference (Δχ^2^; *p* > .05) and a CFI difference lower than 0.01 were the criteria adopted from a step to another in the invariance analysis to determine if the further stricter assumption of invariance was still reasonable.

### Structural model

A two-waves structural equation model with latent variables was tested. Since it was hypothesized that CCB may have a predictive influence on SMS (i.e., mediator), which in turn may influence VC (i.e., outcome), a model with the first-time measured variables (i.e., CCB-T1, SMS-T1, VC-T1) as exogenous and the second-time measured variables (i.e., CCB-T2, SMS-T2, VC-T2) as endogenous was specified (Fig. 1). In particular, each endogenous variable was regressed: (a) on the same variable measured at T1 (i.e., autoregressive path), (b) on the variable(s) hypothesized as antecedent(s), measured at T1. Thus, a structural path from CCB to SMS-T2 (i.e., mediation path a, H1a) was specified, as well as one from SMS-T1 to VC-T2 (i.e., mediation path b, H1b). Since it was a two-waves model (Cole and Maxwell, 2003), the indirect effect was computed as the product of the mediation paths, a*b (MacKinnon, 2012). It was used a bootstrapping procedure (95% CI, 10,000 resamples) to estimate the confidence interval of each parameter of the tested models (Cheung, 2009; MacKinnon, 2012). The indirect effect was considered statistically significant at the *p* < .05.

A model with the endogenous variables’ role inverted (VC as mediator, SMS as outcome) was tested and compared with the former. Next, a model similar to the former was specified by adding some control variables (i.e., Age, household income) as predictors of mediator and outcome variables (i.e., SMS, VC).

A case influence analysis was performed to verify the presence of potential influence cases on model fit by means of the leave-one-out likelihood distance approach (Cook & Weisberg, 1982). After having scrutinized possible similarities among influence cases, the former model was performed excluding them.

## Results

### Preliminary analysis

All participants completed the whole survey in both measurement occasions, neither nonresponse nor attritions were observed. The correlations among all observed variables were statistically significant and ranged from |0.12| to |0.70|. No concern of multicollinearity among all observed variables emerged from the multiple regression with a fictitious outcome variable. The Variance Inflation Factor (VIF) of each predictor was lower than 2 (Dormann et al., 2013).

### Reliability, common method bias and parceling

The McDonald’s ω of each scale (in both the measurement occasions) was greater than the widely assumed cut-off of 0.70 for an acceptable internal consistency (Table 1). Only the CCB showed a McDonald’s ω of 0.61, that is just acceptable, due to the heterogeneity of the content of the few items. The AVE index shown by each scale was higher than the generally assumed threshold of 0.50 (Fornell and Larcker, 1981), thus offering evidence of convergent validity. As well as for the McDonald’s ω the CBB showed an AVE index between 0.35 and 0.40 for the same above-mentioned reasons.

The Harman’s single-factor test suggested an absence of common method bias. Indeed, the CFA with a single latent factor provided worse fit indices: χ^2^(209) = 1331.892, *p* < .001; χ^2^/*df* = 6.37; CFI = 0.97; RMSEA = 0.100, 90%CI: 0.094–0.105, p(RMSEA < .05) < .001; SRMR = 0.121) than the abovementioned CFA with the correlated latent factors: χ^2^(199) = 291.952, *p* < .001; χ^2^/*df* = 1.46; CFI = 1.00; RMSEA = 0.02, 90%CI: 0.022–0.036, *p*(RMSEA < .05) = 0.99; SRMR = 0.038). Model comparison suggested the absence of common method bias: ΔSB χ^2^(10) = 1039.9, *p* < .001.

### Measurement model and invariance analysis

The preliminary CFAs and the comparisons between different specified models suggested the unidimensionality of the respective scales.

The Invariance Analysis suggested a strong invariance for the SMS, and a partial-strong invariance was reached both for CCB and VC. Particularly, for CCB one indicator on 3 was found to be an “offending indicator” (Little, 2013), and for VC, 2 parcels out of 5 were “offending indicators”, meaning that had a different intercept thus reaching only a partial-strong invariance (Tables 2 and 3). The final Models, in which strong invariance (for SMS) and partial strong invariance (for CCB and VC) were imposed, showed a good fit (Table 4).

**Table 2:** Invariance analysis

Constructs	Model tested	χ^2^	*df*	Δχ^2^	Δ*df*	*p*	ΔCFI
CCB	Null model	940.482	21	–	–	< .001	–
	Configural invariance	5.71	5	–	–	< .001	–
	Weak invariance	6.583	7	0.87	2	.67	< 0.001
	Partial-Strong invariance	7.348	9	0.84	2	.68	< 0.001
SMS	Null model	1622.90	21	–	–	< .001	–
	Configural invariance	0.11	5	–	–	< .001	–
	Weak invariance	0.58	7	0.47	2	.78	< 0.001
	Strong invariance	1.24	9	0.65	2	.72	< 0.001
VC	Null model	6183.54	55	–	–	< .001	–
	Configural invariance	24.08	29	–	–	.72	–
	Weak invariance	27.89	33	3.81	4	.43	< 0.001
	Partial-Strong invariance	31.16	36	3.27	3	.35	< 0.001

*Note: CCB* COVID-19 conspiracy beliefs, *SMS* Scientifical-medical satisfaction, *VC* Vaccine confidence, *χ*^*2*^ Chi-square, *df* Degrees of freedom, *Δχ*^*2*^ Delta in chi-square, *Δdf* Delta in degrees of freedom, *p* *p*-value, *ΔCFI* Delta in comparative fit index

**Table 3:** Strong invariance models—*Goodness-of-fit* indices

Model	χ^2^	*df*	*p*	χ^2^ /*df*	CFI	RMSEA (90% CI)	*p*(RMSEA < .05)	SRMR
CCB*	7.348	9	0.601	0.81	1.00	< .001 (0, 0.04)	0.98	0.025
SMS	1.242	9	0.990	0.13	1.00	< .001 (0, 0.01)	0.99	0.011
VC*	31.168	36	0.698	0.86	1.00	< .001 (0, 0.02)	0.99	0.032

*Note*: *CCB* COVID-19 conspiracy beliefs, *SMS* Scientifical-medical satisfaction, *VC* Vaccine confidence, *χ2* Chi-square, *df* Degrees of freedom, *CFI* comparative fit index, *RMSEA* Root Mean Square Error of Approximation, *SRMR* Standardized Root Mean Squared Residual, 90% *CI* confidence intervaal at 90%, * stands for partial invariance model

### Structural model

The hypothesized model showed adequate goodness-of-fit indices: χ^2^_(206)_ = 486.846, *p* < .001; χ^2^/*df* = 2.36; CFI = 0.99; RMSEA = 0.050, 90%CI: 0.044–0.056, *p*(RMSEA < .05) = .478; SRMR = . 064.

**Fig. 2:**
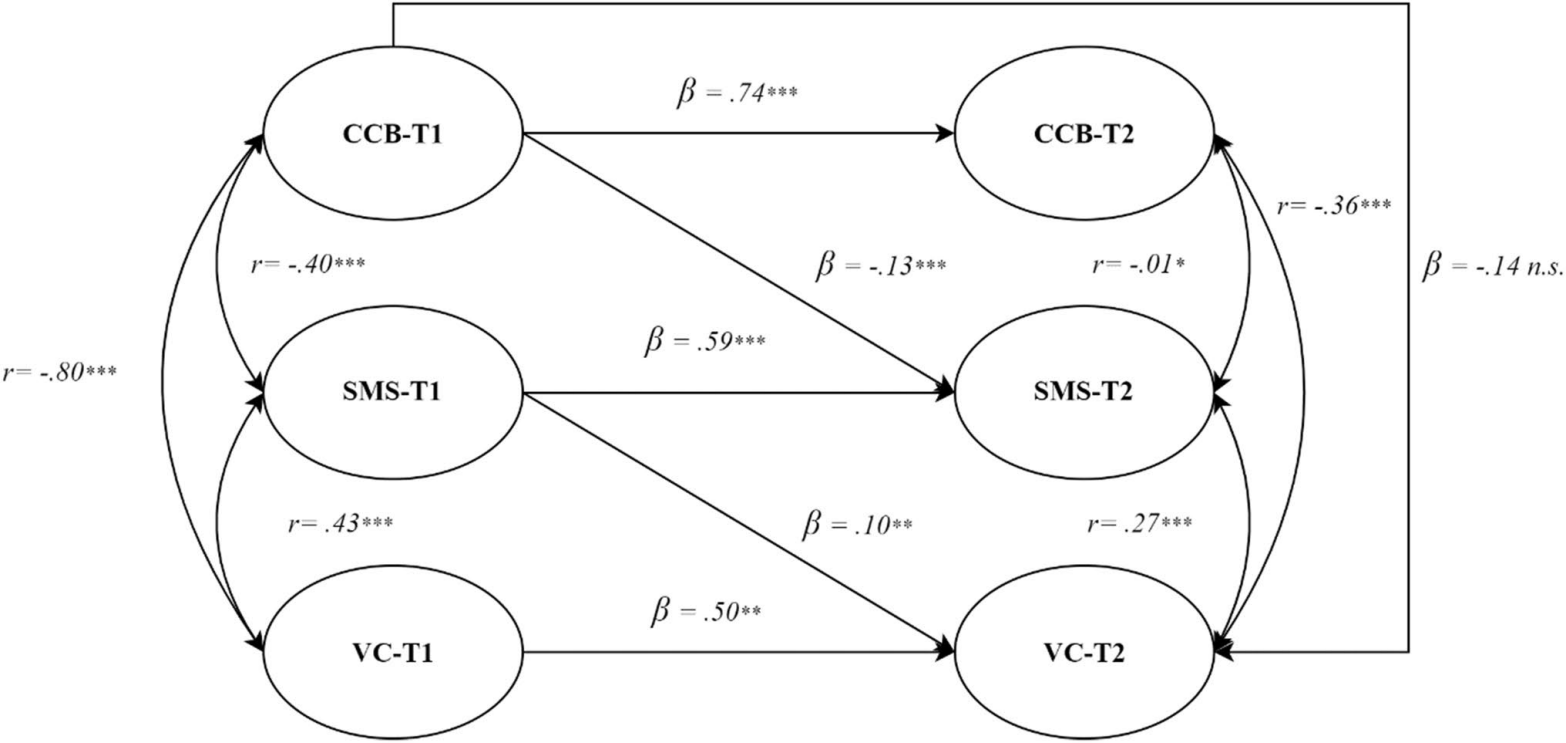
Hypothesized Structural Model. Pearson correlation coefficients = *r*; standardized beta coeefficients = β; * *p* < .05; ** *p* < .01; *** *p* < .001; n.s.: not significant. The measurement model is removed from the diagram for sake of simplicity

From the analysis of the direct effects among the latent variables it emerged that high levels of CCB at T1 were significant predictors of low levels of SMS at T2 (β = − 0.12, SE = 0.03, *p* < .001; H1a), but not of VC at T2 (β =− 0.13, SE = 0.13, *p* = .338; H1c). High levels of SMS at T1 were predictive of high levels of VC at T2 (β = 0.14, SE = 0.05, *p* < .01; H1b). As the direct effect (H1c) from CCB-T1 to VC-T2 is not statistically significant (β = − 0.14, SE = 0.13, *p* = 0.338), it emerged that the relationship between CCB and VC is fully mediated by SMS (β = − 0.02, SE = 0.01, *p* < .05; H2, Table 4). This model explained 42% of the variance of SMS (R^2^ = 0.42) and 43% of the variance of VC (R^2^ = 0.43; Fig. 2).

**Table 4:** Structural model coefficients

Hypothesis	Structural path	*β*	SE	95% CI	*z*	*p*
H1*a*	CCB-T1 → SMS-T2 (*a*)	− 0.13	0.03	[− 0.20,− 0.05]	− 3.20	< .001
H1*b*	SMS-T1 → VC-T2 (*b*)	0.10	0.05	[0.05, 0.23]	2.71	< .01
H1*c*- direct effect	CCB-T1 → VC-T2	− 0.14	0.13	[− 0.19, − 0.03]	− 0.95	.338
H2- indirect effect	(a*b)	− 0.02	0.01	[− 0.03, − 0.01]	− 2.01	< .05
Total effect	CCB-T1 → VC-T2	− 0.15	0.13	[− 0.21, − 0.04]	− 1.10	.270
Autoregressive 1	CCB-T1 → CCB-T2	0.74	0.09	[0.95,1.29]	11.169	< .001
Autoregressive 2	SMS-T1 → SMS-T2	0.59	0.06	[0.65, 0.91]	12.31	< .001
Autoregressive 3	VC-T1 → VC-T2	0.50	0.24	[0.55, 0.82]	2.74	< .01

*Note: CCB* COVID-19 conspiracy beliefs, *SMS* Scientifical-medical satisfaction, *VC* Vaccine confidence, *T1* Time 1, *T2* Time 2, *b* Beta coefficient, *β* Standardized beta coefficient, *SE* Standard error, *95% CI* Lower and upper bounds of the 95% confidence interval, *z* z-value, *p*, *p*-value

The alternative model with the endogenous variables’ role inverted (VC as mediator, SMS as outcome), showed slightly worse goodness-of-fit indices: χ^2^_(206) _= 497.090, *p* < .001; χ^2^/*df* = 2.41; CFI = 0.98; RMSEA = 0.058, 90%CI: 0.049–0.066, *p*(RMSEA < .05) = 0.377; SRMR = 0.070), suggesting a lower adaptation to the data and a lower plausibility of the hypothesized relationship (i.e., CCB to VC to SMS).

The model with control variables showed the following goodness-of-fit indices: χ^2^(246) = 830.593, *p* < .001; χ^2^/*df* = 3.37; CFI = 0.98; RMSEA = 0.065, 90%CI: 0.061–0.071, *p*(RMSEA < .05) < .001; SRMR = 0.073. Age was predictive neither of SMS levels at T2 (β = 0.003, SE = 0.01, *p* = .125) nor of VC levels at T2 (β = 0.004, SE = 0.003, *p* = .821). High levels of household income were predictive of lower levels of SMS (β = 0.08, SE = 0.02, *p* < .05) and higher levels of VC (β = 0.086, SE = 0.03, *p* < .01) at T2. However, effect size was extremely small or negligible (Ferguson, 2009). There was not an appreciable change concerning the explained variance of the endogenous variables (ΔR^2^ < 0.001, for both).

The influence analysis highlighted the presence of 9 influential cases. There was no evidence of similarities among them. After deleting these cases from the estimate of the hypothesized model, no appreciable better fit emerged.

## Discussion

Despite the impact of COVID-19 on the physical and psychosocial spheres, a number of people are still reluctant to get vaccinated, and this is a problem for the containment of the disease and its consequences.

This longitudinal study aimed at understanding the relations between conspiracy beliefs that could lower (i.e., negative association with) the vaccine confidence.

As a first step, the measurement invariance analysis showed that SMS reached strong invariance, CCB and VC reached a partial-strong invariance, indicating that these tools can be used to measure the respective constructs in a reliable and consistent way over time.

After ascertaining the measurement invariance, in the second step it was hypothesized that PESM may play a key role in this relationship: conspiracy beliefs should negatively influence the SMS, which in turn should positively influence vaccine confidence. Results of the two-waves model successfully confirmed the hypothesized pattern. Indeed, the CCB at T1 were significantly and negatively associated (β = − 0.13) with the SMS at T2. In turn, the SMS at T1 was significantly and positively associated (β = 0.10) with VC at T2. Thus, the suggested path leading from CCB, to SMS, to VC is plausible and statistically significant. Interestingly, SMS fully mediated the negative relationship leading from CCB to VC—with a non-statistically significant direct effect (CCB to VC) and a statistically significant indirect effect (CCB to SMS to VC).

The above relationship among variables is the most plausible both from a theoretical and a statistical point of view since the alternative model with the endogenous variables’ role inverted CCB to VC to SMS showed worse fit indices than the one with CCB to SMS to VC.

Also, the model including the control variables showed that age did not influence nor SMS at T2 nor VC at T2, whilst higher household income showed negligible—but still statistically significant—association with lower SMS and thus with higher levels of VC, suggesting how this relationship may deserve further deepening.

From a theoretical point of view, these findings provide a possible explanation on the relations among the factors leading to vaccine confidence and are in line with current literature (Pivetti et al., 2021; Sturgis et al., 2021). From a clinical perspective, these findings provide useful insights for clinical practice given that psychosocial interventions could be planned by considering this path from CCB, to SMS, and VC. For example, some large-scale interventions could target the PESM that has a full-mediator role between the CCB and VC.

Concerning the SMS, it should be the target of psychosocial intervention aimed to raise vaccine confidence, since it proved to fully mediate the relationship from CCB to VC. Thus, rather than trying to modify the rigid CCB, SMS could be a more useful target that can also be more easily modified by encouraging informative campaigns to sensibilize people and spread scientific information through the information channels, the media, and/or the social networks. To this extent, it is important that scientific divulgation is transmitted in a comprehensible and accessible way that makes it feel as more close and ‘familiar’ to people—thus not distant and different.

Regarding the CCB, their effect on VC resulted to be fully mediated by SMS. Also, the cons of CCB as a target of psychosocial interventions include the fact that they are very difficult to modify mostly due to their un-falsifiability. Moreover, literature highlighted how the social media are a preferential channel for the diffusion of CCB—also in our study the correlation between CCB and the trust in social media was *r* = 0.26, *p* < *.001*—despite managing the social media contents and use is difficult, this could be a first step to change how CCB are spread without attacking them directly. Once again, the social media may be used to vehiculate a different perspective toward science and medicine rather than directly attacking the conspiracy beliefs.

Some limitations of this observational study should be acknowledged. The tested model was a two-waves, performed on data retrieved in two waves rather than three. Future studies will test a longitudinal model on three time points. Another point to highlight is the correlation of *r* = 0.80 between the latent factors of conspiracy beliefs and vaccine confidence—however, such value is below the suggested cut-off of 0.84 for multicollinearity that did not emerge nor in the observed variables with the VIF indices (Consoli et al., 2020; Tabachnick & Fidell, 2007). Also, some scales were created ad hoc by adapting some pre-existing tools from other countries (McBride et al., 2021), nonetheless they showed good psychometric properties. About the sampling procedure, the sample was not retrieved though a probabilistic method, despite some criteria were chosen to match with the population characteristics (e.g., age, gender, income, region).

Future studies should also consider in the model the variables that could have an influence on the measured constructs, such as trust, right-wing authoritarianism, the perception of the world as dangerous, the need to control events and intolerance of uncertainty (Freeston et al., 2020) generating the urge to reduce uncertainty and to develop (conspiracy) theories to explain the complexity of reality (Jost et al., 2004). An experimental design is needed to confirm the findings of this observational study.

This study has several strengths, such as the rigorous methodology, the sample characteristics of the Italian population—age, gender, income, region (Bruno et al., 2020; Panzeri, Bertamini, et al., 2021; Panzeri, Rossi Ferrario, et al., 2021), the longitudinal design, the partial-to-strong measurement invariance, the theoretically based model, as well as the importance of the topic for the current issues of the society. Moreover, the innovation of this study relies in targeting the satisfaction with science and medicine—since this is an overlooked but important construct to assess.

These findings could be interesting at a societal level with practical consequences both on the psychological and societal perspectives, with the aim to raise the vaccination rates. Moreover, they can be applied not only to the COVID-19-context but also to other infectious diseases since the negative attitudes towards vaccines are a well-known dynamic—old as vaccines themselves—showing up each time that a new disease/vaccine emerges (e.g., HPV, Zika, smallpox).

In conclusion, this study highlights how conspiracy beliefs about COVID-19 can lead to a lower scientifical-medical satisfaction which in turn represent a full-mediator that can negatively influence the vaccine confidence of individuals. More than buffering the conspiracy beliefs, improving the scientifical-medical satisfaction could be a more useful strategy to raise the vaccine confidence of individuals, thus contributing to improving vaccination rates for everyone’s health.

## Data Availability

The data is not available to protect the participants privacy.

## Code availability

The code is not available.
